# The Effects of Strain-Based Work–Parenting Conflict on Dual Income Couples’ Energy

**DOI:** 10.3390/ijerph19159125

**Published:** 2022-07-26

**Authors:** Jensine Paoletti, Jaye L. Derrick, Christopher P. Fagundes, Kenneth E. Leonard

**Affiliations:** 1 Department of Psychological Sciences, Rice University, Houston, TX 77005, USA; christopher.fagundes@rice.edu; 2 Department of Psychology, University of Houston, Houston, TX 77004, USA; jlderrick@uh.edu; 3 Department of Behavioral Science, University of Texas MD Anderson Cancer Center, Houston, TX 77030, USA; 4 Department of Psychiatry, Baylor College of Medicine, Houston, TX 77030, USA; 5 Clinical and Research Institute on Addictions, University at Buffalo, Buffalo, NY 14260, USA; kleonard@buffalo.edu

**Keywords:** parenting, work–family conflict, strain, energy, actor–partner interdependence model

## Abstract

(1) Background: Gender differences between men’s and women’s parenting roles are well-documented as the “second shift”. We examined the main effects and interaction of work distress and parenting distress with energy (i.e., vigor) in a sample of 310 dual-income, different-sex couples with kids married for approximately nine years. (2) Methods: We used actor–partner interdependence modeling (APIM) to examine how spouses’ distress was associated with their energy. (3) Results: For both wives and husbands, there were negative associations between the actor’s parenting distress and their energy level and between the actor’s work distress and their energy level. However, only wives experienced a significant interaction of work and parenting distress such that high levels of both forms of distress were associated with low levels of energy, indicating that only wives experience this form of work–family conflict. (4) Conclusions: When women experience more strain at home than men, they may need more time to recover from their work and family duties. If they cannot do so, they will have less energy to carry out their responsibilities and may be at a higher risk of future adverse health outcomes.

## 1. Introduction

Before most working women reach the ‘glass ceiling’ blocking them from top leadership positions, they must contend with the ‘maternal wall’, a set of stereotypes and discriminatory barriers for working mothers [1,2]. Working mothers must face benevolent sexism, such as underestimations of work commitment [3,4]. At home, women’s role as the primary caregiver for their children is often driven by the prescriptive stereotypes that assign childrearing as a primarily maternal responsibility [5,6,7]. During the coronavirus pandemic, many working mothers reduced work hours or left the workforce entirely to parent full-time when daycare was unavailable and alternative childcare arrangements were difficult to access [8,9]. Mothers in the workforce must contend with the ‘second shift’ if their male partners do not increase their involvement in childcare and other unpaid household tasks [5,10]. The second shift of unpaid parenting and domestic labor after a workday also has the potential for far-reaching adverse health effects.

Childcare is an effortful nonwork demand that proceeds allostatic load [11,12]. Work-related stress, which may arise from various sources (e.g., low job control or inadequate resources to meet job demands), is also a persistent antecedent to allostatic load [11,13,14]. The allostatic load model indicates that adverse effects on health result from the cumulative effects of stressors, including psychosocial stressors like work or parenting [15,16]. Chronic overactivation of the physiological stress response system without sufficient opportunities for recovery may lead to allostatic overload and related disease endpoints such as cardiovascular disease, type II diabetes, and dementias [14,15,17,18,19,20,21,22,23].

Recovery from daily strains may help reduce the allostatic load; recovery occurs when a person is no longer exposed to the stressor, and their load reactions are alleviated, such as when an employee returns home and engages in a low-stress activity [24]. However, when employees come home from a day of work to parenting duties, they are less likely to recover from work [25]. According to a recent meta-analysis, childcare and related domestic duties are positively correlated with employee fatigue and negatively correlated with after-work relaxation [25]. When parenting burdens primarily fall on working mothers, rather than being split equally between mothers and fathers, we expect mothers to experience short-term and long-term adverse health effects.

In the present study, we examine the strains associated with parenting and work and how they affect working parents’ energy levels. Specifically, we use actor–partner interdependence modeling (APIM) to analyze the association between both parents’ (1) work-related distress and energy, (2) parenting-related distress and energy, and (3) the interaction between work and parenting distress and energy. We argue that the interaction of work and parenting distress provides a valuable measure of work–family conflict. We are interested in studying how these variables affect energy because it reflects one’s capacity to engage the sympathetic nervous system to meet demands; thus, low levels of energy are considered a risk factor for allostatic overload [17,26,27,28]. Ultimately, our goal is to examine the intersection of gender, work distress, and parenting distress as an early indicator of health risk. Many researchers have examined the associations between work–family conflict and health outcomes, including several recent reviews [25,29,30], but fewer studies apply a dyadic perspective. Additionally, it is not common for researchers engaging in work–family literature to take an allostatic overload perspective on health outcomes. Therefore, the present study has three main contributions to the literature: first, we use a dyadic approach to study working parents’ health; second, we employ an allostatic overload perspective; and third, we provide contextual moderators of interdependence theory.

### 1.1. Parenting Distress

Childcare is taxing, effortful work and may increase one’s allostatic load [11,12]. Recent research indicates that stress related to parenting may mirror work-related stress; just as employees may experience burnout, parents may also experience burnout [31,32]. Parental burnout is characterized by exhaustion in taking care of children, emotional distancing from children, and low feelings of personal accomplishment with regard to parenting; parental burnout is positively correlated to depressive symptoms [31,32]. Therefore, we expect both parents will have a negative association between their parenting distress and their energy. In addition, we expect that wives will have higher levels of parenting distress. Prior research suggests that women are likely to perform the most demanding components of childcare (e.g., providing meals, bathing, putting children to bed), while men’s role in childcare involves more play [33]. Similarly, women are more likely than men to use highly involved parenting styles [34]. Therefore, wives’ parenting experiences should feel more distressing relative to their husbands’ parenting experiences.

**Hypothesis** **1** **(H1).** *For (H1a.) wives and (H1b.) husbands, there is a negative association between their own parenting distress and their own energy level. There is (H1c.) a gender difference between husbands and wives, such that wives will have higher levels of parenting distress.*

### 1.2. Work Distress

Work-related stressors are attributed to an estimated $125 billion in increased healthcare costs among Americans alone [35]. There is a large body of research investigating the various components of work, and related boundary conditions, that result in employee strain. The demands associated with work are significantly associated with physical and psychological strain [36]. Therefore, for all participants, we expect a negative association between one’s work distress and one’s energy. We do not expect gender differences in work distress because meta-analytic research suggests that men and women have equal levels of work-related stress [37].

**Hypothesis** **2** **(H2).** *For (H2a.) wives and (H2b.) husbands, there is a negative association between their own work distress and their own energy level.*

### 1.3. Work–Parenting Conflict

Strain-based work–family conflict occurs when stress from one domain makes it challenging to fulfill the requirements of the other role [38]. Work–family conflict is the interaction of work and nonwork experiences; we will examine the interaction of parenting distress and work distress as our measure of strain-based work–parenting conflict. Recall that work and family can be sources of stress that adversely impact energy levels. When parents feel their work life and home life are incongruent, such that they cannot accomplish their tasks from both domains, we expect a negative synergistic effect on their energy levels. Our hypothesis mirrors findings on related health outcomes due to work–family conflict [25,29,30]. Additionally, we expect a gender difference such that women have higher work–parenting conflict for several reasons. First, working mothers must contend with disparate expectations in two domains, as stereotypes of a good parent and good employee are less congruent for women than for men [39]. Likewise, men’s work–family conflict is reduced when they have social support from their coworkers, yet the same is not true for women [40]. Finally, meta-analytic findings also demonstrate that mothers have higher work–family conflict than fathers [41].

**Hypothesis** **3** **(H3).** *For (H3a.) wives and (H3b.) husbands, there is an interaction between actor parenting distress and actor work distress on energy such that high levels of both forms of distress are negatively associated with actor energy. There is (H3c.) a gender difference between husbands and wives, such that wives will have higher levels of the work–parenting distress interaction.*

## 2. Materials and Methods

### 2.1. Sample and Procedure

Data was collected as part of a larger, longitudinal study of about 650 couples who were married between 1996 and 1999. Couples were recruited at the city courthouse when applying for their marriage licenses. At the time of recruitment, participants were at least 18 years old, spoke and read English, and had never been married previously (for more information about recruitment, see Homish & Leonard, 2007) [42]. Couples were surveyed periodically (e.g., first, second, fourth, seventh, and ninth anniversaries) throughout their marriage about their health behaviors, including drinking and substance use behaviors. The data for the present study was collected during the sixth wave of the longitudinal study, between September 2005 and September 2008, at the couples’ ninth anniversary. The sixth wave of data collection efforts expanded to include work-related and parenting-related questionnaires to gain a better understanding of the various stresses that couples experience.

We used the sixth wave of the larger study only because prior waves did not include measures of work experiences. We had data from 318 couples. Our interest in the work–parenting dynamics led us to create additional inclusion criteria for our analyses, such that we only included couples in which both spouses are parents. However, there are only eight couples in which only one spouse is a parent. Similarly, all included participants worked outside the home. Our final sample includes 310 different-sex couples of working parents, with 310 husbands and 310 wives for a total of 620 participants. Many people in our sample have two children and work about 50 h per week (see Table 1 for sample characteristics).

### 2.2. Materials

#### 2.2.1. Parenting and Work Distress

Distress in parenting and distress at work were measured with variations of the same six-item scale [43]. Participants were asked to think about the daily pleasures and problems in their role as a parent or employee, respectively, and indicate the extent to which they feel various emotions. Specifically, participants responded to items about how bothered or upset, relaxed, frustrated, fortunate, unhappy, and pleased they feel on a four-point scale (“very”, “somewhat”, “only a little”, or “not at all”). The positively worded items were reverse coded; the scale was mean scored at the pooled husband–wife mean and centered. We had adequate internal consistency for parenting-related distress (α = .83 for wives and α = .82 for husbands) and work-related distress (α = .84 for wives and α = .86 for husbands).

#### 2.2.2. Energy

Energy was measured with the vigor/energy subscale of the Short-Form Health Survey (SF-36) [44]. Participants answered four items about their experiences with fatigue and energy for the last four weeks on a six-point scale (e.g., “Did you have a lot of energy?” answered with “all of the time”, “most of the time”, “a good bit of the time”, “some of the time”, “a little of the time”, or “none of the time”). Standardized scores range from 0 to 100, with higher scores indicating more energy. We had adequate internal consistency (α = .84 for wives and α = .80 for husbands).

#### 2.2.3. Marital Satisfaction

Marital satisfaction was measured using the Marital Adjustment Test [45]. The measure was included as a covariate to examine the effects of work and parenting distress while accounting for the established correlations between marital satisfaction and similar constructs with our variables of interest [40,46]. Participants responded to 15 items about conflict, leisure, and relationship quality (e.g., “What is your degree of happiness, everything considered, in your current relationship?”). The scale was summed and centered at the pooled husband–wife mean. We had good internal consistency (α = .97 for wives and α = .96 for husbands).

#### 2.2.4. Data Analysis

To test the hypotheses, we ran an actor–partner interdependence model (APIM) using structural equation modeling (SEM) with maximum likelihood estimation in the lavaan package in R [47,48]. Modeling with SEM for APIM allows for two simultaneous equations, one for each member of the couple. This model accounts for the nonindependence in the associations between predictors and outcome variables by correlating all exogenous variables and disturbances across members of the couple. APIM allows for an examination of actor effects (e.g., the association between husbands’ work distress and husbands’ energy) as well as partner effects (e.g., the association between husbands’ work distress and wives’ energy). Prior to analysis, predictor variables were centered at the pooled mean for husbands and wives. We ran a model with the main effects (i.e., work distress and parenting distress) and another with main effects plus an interaction term of work distress and parenting distress. The results of the main effects model and the interaction model may be found in Table 2. We added marital satisfaction as a covariate in both models; therefore, the distress variables are endogenous [47]. To test for gender differences, we started with a just-identified model and constrained the paths to be equal across husbands and wives; this procedure creates 1 degree of freedom, allowing a chi-square test to compare the original, unconstrained model and the gender-constrained model [49]. If the chi-squared test indicates a significant difference between the unconstrained and constrained models such that the unconstrained model is a better fit for the data, then there are significant gender differences in the tested path [49].

## 3. Results

**Figure 1:**
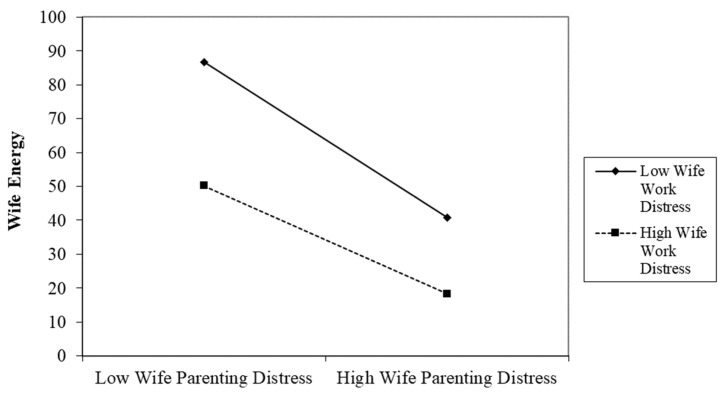
Interaction of wives’ work distress and parenting distress. Low and high levels of distress are one standard deviation below or above the mean, respectively.

Descriptive statistics on study variables and participant demographic variables, plus one-way ANOVA comparisons between husbands and wives on variables, can be found in Table 1. The results of the APIM analyses can be found in Table 2 and Figure 1. Intercorrelations between study variables can be found in Appendix A. The intraclass correlation (ICC) was calculated using the ANOVA method. The ICC was .22, indicating that about 22% of the variance in energy was attributable to the dyadic relationship between husband and wife.

In our APIM with main effects only, we found support for Hypothesis 1a and 1b, as there was a negative association between actor parenting distress and energy both for wives (*B* = −14.46, *SE* = 2.06, *p* < .001) and husbands (*B* = −1.50, *SE* = 2.24, *p* < .001). We found support for H1c, by comparing the hypothesized model in which parenting distress actor effects are different for husbands and wives with a constrained model in which husbands and wives had the same parenting distress actor effect. The hypothesized model with separate husband and wife actor effects had a significantly better fit than the constrained model (Δχ^2^(1) = 8.19, *p* = .004), indicating that the husband’s parenting actor effect is significantly different from the wife’s parenting actor effect. We found support for Hypothesis 2a and 2b, as there was a negative association between actor work distress and energy both for wives (*B* = −7.12, *SE* = 2.25 *p* = .002) and husbands (*B* = −5.64, *SE* = 2.06, *p* = .006).

Beyond the hypothesized associations, we found no significant partner effects. However, the actor effect of husbands’ marital satisfaction was significantly associated with husbands’ energy; wives’ marital satisfaction was unrelated to wives’ energy. As an exploratory analysis, we compared the hypothesized model in which work distress actor effects are different for husbands and wives with a constrained model in which husbands and wives have the same work distress actor effect. The hypothesized model with separate husband and wife actor effects had a significantly better fit than the constrained model (Δχ^2^(1) = 7.21, *p* = .007), indicating that the husband’s work actor effect is significantly different from the wife’s work actor effect.

In our APIM with interaction terms between parenting distress and work distress, we found support for Hypothesis 3a, as there was a negative association between the interaction term of parenting distress and work distress on energy for wives (*B* = 1.21, *SE* = 3.98, *p* = .010). We probed the two-way interaction between wives’ parenting distress and wives’ work distress, and we found a significant slope at low levels of wife work distress (i.e., one standard deviation below the mean; simple slopes test, *B* = −4.46, *p* < .001) and high levels of wife work distress (i.e., one standard deviation above the mean; *B* = −28.01, *p* < .001). We did not find support for Hypothesis 3b, as there was no significant association between the husbands’ distress interaction term on husbands’ energy.

We found support for H3c by comparing the hypothesized model in which the actor effect for the distress interaction term effects is different for husbands and wives with a constrained model in which husbands and wives had the same distress interaction term. The hypothesized model with separate husband and wife actor effects had a significantly better fit than the constrained model (Δχ^2^(1) = 6.96, *p* = .008), indicating that the husband’s distress interaction term is significantly different from the wife’s distress interaction term. Although not hypothesized, there was a significant actor effect of husbands’ marital satisfaction on husbands’ energy (*B* = .12, *SE* = .04, *p* = .006), but no significant corresponding association between wives’ marital satisfaction and wives’ energy.

## 4. Discussion

We investigated the intersection of two important domains (work and family) on an early indicator of future health (energy) [17,26,27,28,50]. For husbands and wives, we found negative associations between work distress and energy and between parenting distress and energy. We also found an interaction between work and parenting distress for wives’ energy, but no such interaction for husbands. For wives, parenting distress attenuates the negative association between work distress and energy. Parenting distress and work distress have an additive effect such that wives have lower levels of energy when they have high distress in both domains; this finding indicates that women’s parenting and work experiences *together* have a negative correlation with her energy levels. Finally, we found that wives had higher levels of parenting distress and higher levels of the parenting–work distress interaction (i.e., work–parenting conflict). Overall, our findings indicate that, although all working parents experience negative health outcomes from work and parenting distress individually, working mothers may disproportionally suffer from work–parenting conflict. Working mothers experience more strain while parenting than their spouses and have lower energy levels when both parenting and work are highly distressing. Over time, women’s allostatic load may be adversely affected by their work–parenting conflict.

The findings from our study align with the broader literature, as women’s higher levels of work–family conflict are thought to be driven by their greater number of hours doing domestic work relative to men [41]. While we did not capture relative hours of parenting in our study, women did find their parenting experiences more distressing than men. There is a persistent expectation of mothers as the more involved parent that women and men may both internalize [5,7]. For instance, mothers may exhibit maternal gatekeeping behaviors, such that they are reluctant to relinquish responsibility for family matters, often because of the centrality of motherhood to their identity [51]. Mothers’ gatekeeping behavior inhibits fathers’ ability to be involved parents and increases their own parenting burden. In fact, researchers have found that women’s job flexibility may encourage maternal gatekeeping and lead to her subsequent work–family conflict [52].

While our findings are consistent with prior research, there are alternative explanations. We did not find an interaction effect between fathers’ work distress and parenting distress on their energy levels. While we attribute this effect to lower strain-based work–family conflict for fathers relative to mothers, it is also possible that there is a floor effect such that fathers’ work–parenting interaction is unobservable relative to mothers’ work–parenting interaction. Other findings suggest that, compared with men, women experience lower recovery at the end of a workday, indicating that women are likely to have higher work–family conflict [53,54]. Women’s lower levels of recovery from work (and perhaps from family too) may explain how women’s higher work–family conflict is related to their energy levels, as recovery promotes energy [55]. Recovery occurs when exposure to stressors has ended, but if stress from work ‘follows’ women home via work–family conflict, there may be inadequate opportunity for recovery. Over time, inadequate recovery can increase the risk of allostatic overload. Alternatively, the association between women’s work–family conflict and energy levels may be driven by other factors, as there is some evidence for gendered effects in stress-coping mechanisms and their underpinnings [56,57,58].

### 4.1. Theoretical Applications

Interdependence theory addresses the mutual dependencies between individuals, especially between married couples. Mirroring most of the prior work–family literature, our hypotheses focused on actor effects in the APIM analyses. Indeed, we found evidence for actor effects, but not partner effects in our specific context of dual-income parents. There is a plethora of work to show coping is an interdependent dyadic process, which results in partner effects, including those that suggest that men impact women’s experiences more than women impact men’s [59,60]. However, there are certain contexts where actor effects may be much stronger than partner effects [61,62]. It is possible that we found no evidence for partner effects because couples are as interdependent in the workplace as they are in other contexts. There is some evidence that people interpret their work experiences as independent [63,64], but perhaps parenting is also interpreted as an independent experience. Working mothers find parenting more distressing than working fathers, perhaps because they take on a disproportionate parenting load.

### 4.2. Practical Applications

Practically, the results of our study suggest that working mothers, especially those with high parenting and work distress, are at a higher risk of allostatic overload and related adverse health outcomes if their distress remains chronically high. The work recovery literature may provide valuable insights into how working parents can reduce their risk of adverse health outcomes. Working parents may experience recovery when they psychologically distance themselves from work [54,64,65]. They may also experience recovery through mindfulness, exercise, and verbalizing their emotions [54,64,66]. The role of choice may also be important; if parents can minimize household activities (e.g., cooking and cleaning) on days they do not want to do those activities, then they may increase their recovery [67]. Of course, there are gendered, structural challenges that make it challenging for women to minimize their household activities; as suggested by others, men may contribute more to household activities [68]. Finally, receiving support from a spouse on the above strategies may increase recovery and life satisfaction [69]. In particular, working fathers should provide support to their spouses to help address working mothers’ work–parenting conflict.

### 4.3. Limitations and Future Directions

Our study utilized a sample of couples married for nine years. As half of all divorces occur within the first seven years of marriage, the couples in our sample have passed one major milestone for marital dissolution [70,71]. The couples in our study are middle-aged and likely in mid-career, and thus at the stage of life considered important for career advancement or the “mommy track”, when working mothers’ careers stagnate because of the incompatibility between their work and family lives [72,73,74]. A spouse’s health is thought to converge over time as a result of shared behaviors and experiences, among other factors [59,75].

While the present study has strengths, there are limitations that future research may address. First, the cross-sectional nature of our data impedes the ability to draw causal inferences in the associations examined. For instance, we cannot assert that higher work–family conflict causes lower energy, although meta-analytic research of similar constructs indicates support for a reciprocal effect such that causality goes both ways [76]. Next, our sample is relatively homogeneous, consisting of American, dual-income, two-parent homes. Future research may investigate how couples’ work–family management strategies are related to health. Similarly, research should address how organizations’ flexible work policies and family leave policies benefit employees’ health and the health of their families. Next, as our findings are thought to be driven by cultural factors, such as gender stereotypes around family roles, future research should continue to investigate how gender stereotypes and other cultural factors affect the association between work–family conflict and health outcomes. Work–family conflict is correlated with components of national culture, including gender egalitarianism [77]. Notably, many countries high in gender egalitarianism also have robust social safety nets and national healthcare systems [77]. Valuable future research may include detangling the associations between gender egalitarianism, work–family conflict, and health.

## 5. Conclusions

Family and work are often cited as the two most important social domains [50]. This may be especially true for dual-income couples in mid-career stages, who must balance raising children with their employment. Among these couples, we find that, when accounting for their marital satisfaction, experiencing distress in either domain is related to lower energy levels. However, we found evidence for a form of work–family conflict, which we call work–parenting conflict, among women but not men. This gender difference mirrors other findings in the literature on gendered workplace barriers, yet we focus on the potential adverse health outcomes. Working mothers face barriers to equality at work; we find they may also face obstacles to quality of life.

## Figures and Tables

**Table 1:** Sample characteristics.

Variable	Count (%) or Mean (SD)			
	Overall (*N* = 620)	Wives (*n* = 310)	Husbands (*n* = 310)	*p*-Value
Age	37.22 (6.03)	36.34 (5.66)	38.15 (6.28)	<.001 ***
Race ^a^	–	–	–	.54
*Black*	173 (27.90%)	82 (26.45%)	91 (29.35%)	–
*Asian American*	4 (.65%)	2 (.65%)	2 (.65%)	–
*White*	417 (67.26%)	214 (69.03%)	203 (65.48%)	–
*Hispanic*	18 (2.90%)	9 (2.90%)	9 (2.90%)	–
*American Indian*	5 (.81%)	2 (.65%)	3 (.97%)	–
*Other*	3 (.48%)	1 (.32%)	2 (.65%)	–
Individual Income ^a^	–	–	–	<.001 ***
*Less than $10,000*	28 (6.67%)	23 (11.73%)	5 (2.23%)	–
*$10,000–$19,999*	52 (12.38%)	35 (17.86%)	17 (7.59%)	–
*$20,000–$29,999*	52 (12.38%)	33 (16.84%)	19 (8.48%)	–
*$30,000–$39,999*	69 (16.43%)	30 (15.31%)	39 (17.41%)	–
*$40,000–$54,999*	94 (22.38%)	45 (22.96%)	49 (21.88%)	–
*$55,000–$74,999*	66 (15.71%)	15 (7.65%)	51 (22.77%)	–
*$75,000 and greater*	59 (14.05%)	15 (7.65%)	44 (19.64%)	–
Number of Children	2.44 (1.56)	2.46 (1.66)	2.42 (1.45)	.77
Hours Worked Weekly	5.48 (22.21)	5.59 (26.09)	5.37 (16.98)	.91
Life Satisfaction	24.72 (7.21)	24.76 (7.40)	24.67 (7.02)	.88
Marital Satisfaction	107.21 (27.26)	107.46 (26.92)	106.95 (27.68)	.83
Work Distress	1.95 (.61)	1.99 (.61)	1.93 (.61)	.34
Parenting Distress	1.69 (.56)	1.76 (.57)	1.62 (.54)	.003 **
Energy	55.01 (2.49)	5.87 (21.24)	59.59 (18.62)	.08

Note: *p*-values derived from one-way ANOVA except where otherwise specified. ^a^ indicates *p*-value derived from Wilcoxon–Mann–Whitney test. ** indicates *p* < .01. *** indicates *p* < .001.

**Table 2:** Actor–partner interdependence models on energy.

	Main Effects			Interaction		
Variable	*B*	*SE*	*β*	*B*	*SE*	*β*
	Wives					
Intercept	52.10	1.12	-	52.06	1.18	-
Actor Marital Satisfaction	.08	.05	.11	.08	.05	.10
Partner Marital Satisfaction	.03	.06	.04	.02	.06	.03
Actor Parenting Distress	−14.46 ***	2.06	−.39	−34.47 ***	8.04	−.92
Partner Parenting Distress	1.93	2.85	.05	6.19	7.98	.16
Actor Work Distress	−7.12 ***	2.25	−.21	−24.91 **	7.19	−.72
Partner Work Distress	−3.14	2.40	−.09	.37	6.34	.01
Actor Parenting Distress × Work Distress				1.21 **	3.98	.82
Partner Parenting Distress × Work Distress				−1.88	3.42	−.17
*R* ^2^	.28			.30		
	Husbands					
Intercept	58.58	1.03	-	58.88	1.09	-
Actor Marital Satisfaction	.13 **	.04	.19	.12 **	.04	.18
Partner Marital Satisfaction	−.09	.05	−.13	−.09	.05	−.13
Actor Parenting Distress	−1.50 ***	2.24	−.31	−3.77	6.91	−.11
Partner Parenting Distress	1.12	2.11	.04	−4.03	8.71	−.12
Actor Work Distress	−5.64 **	2.06	−.19	−.45	5.40	−.02
Partner Work Distress	−4.47	2.34	−.15	−9.28	7.90	−.31
Actor Parenting Distress × Work Distress				−3.01	2.95	−.31
Partner Parenting Distress × Work Distress				2.70	4.47	.25
*R* ^2^	.25			.26		

Note: *N* = 310 dyads (620 individuals). ** *p* < .01. *** *p* < .001.

## Data Availability

The data presented in this study are available upon request from the corresponding author. The data are not currently publicly available because we do not have the resources or funding to make the data user-friendly at the level that no PI explanation is required.

## Appendix A

**Table A1:** Correlations between study variables.

Variable	1	2	3	4	5	6	7	8	9	10	11	12	13	14	15	16	17	18
1. W Age	-																	
2. H Age	.77 **	-																
3. W Income	.04	.00	-															
4. H Income	.04	.05	.12	-														
5. W No. of Children	−.03	−.00	−.20 **	−.14 *	-													
6. H No. of Children	.07	.07	−.20 **	−.16 *	.85 *	-												
7. W Weekly Work Hours	−.15 *	−.14 *	.45 **	−.00	.12	.07	-											
8. H Weekly Work Hours	.06	.07	−.07	.29 **	.03	.07	−.05	-										
9. W Life Satisfaction	−.07	−.11	.15 *	.22 **	−.20 **	−.19 **	−.05	−.23 **	(.91)									
1. H Life Satisfaction	−.08	−.06	.03	.30 **	−.10	−.20 **	−.04	−.19 **	.46 **	(.91)								
11. W Marital Satisfaction	−.01	.03	−.10	.14	−.12 *	−.14 *	−.01	−.08	.47 **	.37 **	(.97)							
12. H Marital Satisfaction	−.07	.03	.03	.01	.02	.05	−.05	−.05	.28 **	.59 **	.42 **	(.96)						
13. W Parenting Distress	−.13	−.08	.04	.00	.07	.03	−.07	.05	−.44 **	−.19 **	−.23 **	−.08	(.83)					
14. H Parenting Distress	.01	−.06	−.07	.01	.05	.18 **	−.11	.05	−.18 **	−.40 **	−.18 **	−.32 **	.20 **	(.82)				
15. W Work Distress	−.04	−.09	.12	−.16	.02	.02	.27 **	.01	−.26 **	−.10	−.33 **	−.08	.18 *	.02	(.84)			
16. H Work Distress	−.01	−.02	−.03	.06	−.21 **	−.13	−.18 *	.07	−.08	−.35 **	.05	−.19 **	.04	.40 **	−.02	(.86)		
17. W Energy	.18**	.17 **	−.09	.13	−.05	−.03	−.14 *	−.04	.43 **	.20 **	.27 **	.14 *	−.45 **	−.13	−.30 **	−.07	(.84)	
18. H Energy	.15*	.18 **	.06	.11	−.05	−.13 *	−.02	−.13 *	.11	.37 **	.04	.28 **	−.04	−.42 **	−.09	−.32 **	.12	(.80)

Note: W = wives; H = husbands. Values in the diagonal represent Cronbach’s alpha reliability when applicable. * indicates *p* < .05. ** indicates *p* < .01.
